# Comparison of the applicability of seven calculation equations of glomerular filtration rate among elderly people in China

**DOI:** 10.1007/s11255-024-03941-w

**Published:** 2024-03-11

**Authors:** Weiwei Zhu, Yingyu Zhang, Shutao Chen, Yang Sui, Xufang Wang, Wei Li, Chenxia Juan, Yan Zhou, Kun Gao

**Affiliations:** 1 Division of Nephrology, Affiliated Hospital of Nanjing University of Chinese Medicine, Jiangsu Province Hospital of Chinese Medicine, Nanjing, 210029 Jiangsu China; 2 Division of Molecular Signaling, Department of the Advanced Biomedical Research, Interdisciplinary Graduate School of Medicine, University of Yamanashi, Chuo, 409-3898 Japan; 3 Inheritance Studio of Chinese Medicine Master ZOU Yanqin, Affiliated Hospital of Nanjing University of Chinese Medicine, Nanjing, 210029 Jiangsu China

**Keywords:** Elderly, Estimated GFR, Serum creatinine, Serum Cystatin C, Equation

## Abstract

**Background:**

At present, estimated glomerular filtration rate (eGFR) remains the most frequently utilized parameter in the evaluation of kidney injury severity. Numerous equations have been formulated based on serum creatinine (Scr) or serum cystatin C (Cysc) levels. However, there is a lack of consensus regarding the efficacy of these equations in assessing eGFR, particularly for elderly individuals in China. This study aimed to evaluate the applicability of the MDRD, MDRDc, CKD-EPI series, BIS1, and FAS equations within the Chinese elderly population.

**Methods:**

A cohort of 298 elderly patients with measured GFR (mGFR) was enrolled. The patients were categorized into three subgroups based on their mGFR levels. The eGFR performance was examined, taking into account bias, interquartile range (IQR), accuracy P30, and root-mean-square error (RMSE). Bland–Altman plots were employed to verify the validity of eGFR.

**Results:**

The participants had a median age of 71 years, with 167 (56.0%) being male. Overall, no significant differences in bias were observed among the seven equations (*P* > 0.05). In terms of IQR, P30, and RMSE, the BIS1 equation demonstrated superior accuracy (14.61, 72.1%, and 13.53, respectively). When mGFR < 30 ml/min/1.73 m^2^, all equations underestimated the true GFR, with the highest accuracy reaching only 59%. Bland–Altman plots indicated that the BIS1 equation exhibited the highest accuracy, featuring a 95% confidence interval (CI) width of 52.37.

**Conclusions:**

This study suggested that the BIS1 equation stands out as the most applicable for estimating GFR in Chinese elderly patients with normal renal function or only moderate decline. 2020NL-085-03, 2020.08.10, retrospectively registered.

## Introduction

The etiology of chronic kidney disease (CKD) varies considerably across the world. In high-income and middle-income countries, diabetes and hypertension are the primary drivers of CKD. The global prevalence of diabetes mellitus and hypertension is witnessing a rapid upsurge due to an aging population [1, 2]. Consequently, it is imperative to consistently monitor renal function in the elderly demographic.

At present, the glomerular filtration rate (GFR) serves as the principal indicator for evaluating the severity of kidney damage [3]. Ideally, GFR is measured using an exogenous marker (such as inulin, iohexol, iothalamate, Cr-ethylenediaminetetraacetic acid, etc.) [4]. Nonetheless, measurements employing exogenous markers are often cumbersome and expensive. Consequently, estimated GFR (eGFR) based on a patient's serum creatinine (Scr) and serum cystatin C (Cysc) remains the most widely utilized indicator in clinical settings [5]. Factors, such as gender, age, and muscle volume, can influence Scr [6], while CysC, an endogenous protein, boasts relatively stable production and serum levels not impacted by variables other than renal function [7]. However, Cysc measurements are costly and not routinely conducted in many primary hospitals. As a result, several equations have been developed based on one or both endogenous markers.

In 1999, the MDRD equation was developed by the American Modification of Diet in Renal Disease (MDRD) working group using data from 1070 CKD patients in Western countries [8]. This equation was later simplified by considering four variables: gender, age, creatinine, and race, and became widely used for estimating GFR in Western populations [9, 10]. In 2006, the MDRD equation was modified by the Chinese eGFR Investigation Collaboration to better suit the characteristics of Chinese CKD patients, resulting in the MDRDc equation [11]. However, the MDRD equation tends to underestimate higher levels of GFR. To address this issue, the Kidney Disease/Improving Global Outcomes (KDIGO) organization developed the CKD-EPI creatinine equation in 2009 [12]. This equation, based on serum creatinine values and GFR levels from 5504 participants of diverse ethnicities, including Asians, provides more accurate estimations for both high and low GFR levels. In 2012, the CKD-EPI creatinine-cystatin C equation and cystatin C equation were introduced, incorporating cystatin C as an alternative filtration marker for estimating GFR [13]. Additionally, the Berlin Initiative Study (BIS) equations were introduced in the same year, specifically tailored for elderly individuals [14]. Finally, in 2016, the Full Age Spectrum (FAS) equation was developed to estimate GFR across all age groups [15]. To date, no consensus has been reached regarding the efficacy of these equations in evaluating the eGFR of elderly individuals in China. This study, employing the gold standard GFR measured by 99m-Technetium-diethylenetriamine-pentaacetic acid (^99m^ Tc-DTPA) renal dynamic imaging, aims to assess the applicability of the aforementioned equations in the Chinese elderly population.

## Materials and methods

### Study population and design

**Fig. 1:**
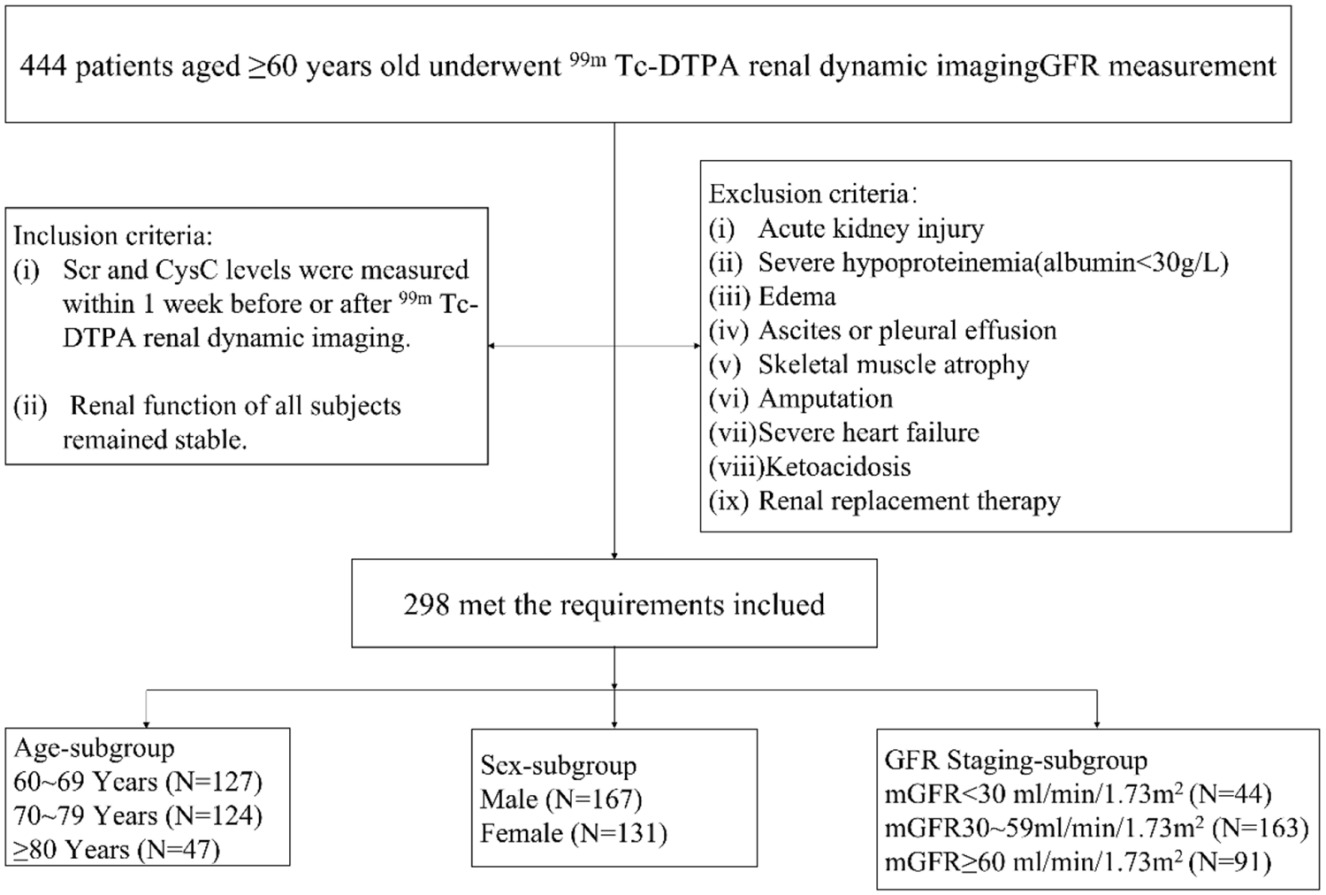
Flowchart of the study

In accordance with Chinese legislation, the age threshold for senior citizens starts at 60 years old [16]. In this comprehensive retrospective study, we enrolled a total of 444 elderly Chinese participants (aged 60 years and above) who were hospitalized at the Division of Nephrology, Jiangsu Province Hospital of Chinese Medicine in China. The study period spanned a decade, from January 2010 to December 2020. All subjects met the diagnostic criteria for CKD as specified in the KDIGO guideline [17] and underwent ^99m^ Tc-DTPA renal dynamic imaging for the measurement of GFR. The following inclusion criteria were used for the subjects: (i) Scr and Cysc levels were measured within 1 week before or after ^99m^ Tc-DTPA renal dynamic imaging; (ii) Renal function of all subjects remained stable. The exclusion criteria were as follows: (i) Acute kidney injury; (ii) Severe hypoproteinemia (albumin < 30 g/L); (iii) Edema; (iv) Ascites or pleural effusion; (v) Skeletal muscle atrophy; (vi) Amputation; (vii) Severe heart failure; (viii) Ketoacidosis; (ix) Renal replacement therapy (Fig. 1). Among the 444 participants, 118 patients were not included due to incomplete Scr or cysc data within a week. Eight patients were excluded due to edema, four patients due to hypoproteinemia, five patients due to CKD with acute kidney injury, one patient due to an amputation, eight patients due to heart failure, and two patients due to pleural effusion. Finally, a total of 298 participants' clinical data were included in this study.

### Laboratory measurements

The Laboratory department of Jiangsu Province Hospital was responsible for all laboratory measurements. Fasting blood samples were collected from the subjects in the morning. The American Beckman Coulter AU5800 automatic biochemical analyzer was employed to measure Scr using the enzymatic method and CysC using latex-enhanced immunoturbidimetry.

### GFR measurements

The gold standard method of renal dynamic imaging was utilized to measure GFR [18]. A single-photon emission computed tomography scanner (SPECT. SKYLIGHT), manufactured by Philips in the Netherlands, was used for the imaging process. The radiotracer employed was ^99m^ Tc-DTPA, which was injected intravenously into the patients, followed by dynamic image acquisition. Post-image processing, the instrument's software calculated the standard GFR, taking into account the patient's weight and height.

### Estimation methods of eGFR

In this study, seven equations (CKD-EPI-Scr, CKD-EPI-Cysc, CKD-EPI-Scr-Cysc, MDRD, MDRDc, FAS, BIS1) were employed to estimate eGFR based on the patients' age, blood Scr, and CysC levels [8–15]. The specific equations used are presented in Table 1.

**Table 1:** The expression of seven equations basd on Scr or Cysc in the study

Year	Equation Name	Equation
1999	MDRD equation	186 × Scr^−1.154^ × age^−0.203^ × (0.742 Female)
2006	MDRDc equation	175 × Scr^−1.234^ × age^−0.179^ × (0.79 Female)
2009	CKD-EPI-Scr equation	144 × (Scr/62)^−0.329^ × 0.993^Age^ (Female,Scr ≤ 62)
		144 × (Scr/62)^−1.209^ × 0.993^Age^ (Female,Scr > 62)
		141 × (Scr/80)^−0.411^ × 0.993^Age^ (Male,Scr ≤ 80)
		141 × (Scr/80)^−1.209^ × 0.993^Age^ (Male,Scr > 80)
2012	CKD-EPI-Cysc equation	133 × (Cysc/0.8)^−0.499^ × 0.996^Age^ × (0.932 Female,Cysc ≤ 0.8)
		133 × (Cysc/0.8)^−1.328^ × 0.996^Age^ × (0.932 Female,Cysc > 0.8)
2012	CKD-EPI-SCr-Cysc equation	130 × (Scr/62)^−0.248^ × (Cysc/0.8)^−0.375^ × 0.995^Age^(Female, Scr ≤ 62, Cysc ≤ 0.8)
		130 × (Scr/62)^−0.248^ × (Cysc/0.8)^−0.711^ × 0.995^Age^ (Female, Scr ≤ 62, Cysc > 0.8)
		130 × (Scr/62)^−0.601^ × (Cysc/0.8)^−0.375^ × 0.995^Age^ (Female, Scr > 62, Cysc ≤ 0.8)
		130 × (Scr/62)^−0.601^ × (Cysc/0.8)^−0.711^ × 0.995^Age^ Female, Scr > 62, Cysc > 0.8)
		135 × (Scr/80)^−0.207^ × (Cysc/0.8)^−0.375^ × 0.995^Age^ (Male, Scr ≤ 80, Cysc ≤ 0.8)
		135 × (Scr/80)^−0.207^ × (Cysc/0.8)^−0.711^ × 0.995^Age^ (Male, Scr ≤ 80, Cysc > 0.8)
		135 × (Scr/80)^−0.601^ × (Cysc/0.8)^−0.375^ × 0.995^Age^ (Male, Scr > 80, Cysc ≤ 0.8)
		135 × (Scr/80)^−0.601^ × (Cysc/0.8)^−0.711^ × 0.995^Age^ (Male, Scr > 80, Cysc > 0.8)
2012	BIS1 equation	3736 × (Scr/88.4)^−0.870^ × age^−0.950^ × (0.82 Female)
2016	FAS equation	107.3/(Scr/62) × [0.988^(age−40)^age > 40 years] (Female)
		107.3/(Scr/80) × [0.988^(age−40)^age > 40 years] (Male)

*Scr (μmoI/L)* Serum creatinine, *Cysc(mg/L)* Cystatin C, *MDRD* Modification of diet in renal disease, *MDRDc* MDRD-China, *CKD-EPI* Chronic kidney disease epidemiology collaboration, *BIS1* Berlin Initiative Study 1, *FAS* Full age spectrum. Serum creatinine expressed as μmol/L, while 1 mg/dl equal to 88.4 μmol/L

### Statistical analysis

Data with normal distribution are expressed as mean ± standard deviation ($\overline{x }$± s), while non-normally distributed data are presented as median (interquartile range [IQR]). IQR and median of difference were employed as assessment indices. The percentage of estimates within 30% of the measured value (P30) was defined as the proportion of eGFR values deviating within 30% of the measured GFR (mGFR), and is often used to evaluate the accuracy of the eGFR estimation equation [19, 20]. Root-mean-square error (RMSE) was also utilized to aid the analysis process. The Bland–Altman plot analysis was employed to describe the mean difference and precision between mGFR and eGFR. Non-parametric rank-sum tests and chi-square tests were used for comparing baseline, bias, and accuracy. A *p*-value < 0.05 was considered statistically significant. All calculations and statistical analyses were conducted using IBM SPSS Software (version 26.0, IBM Corp) and GraphPad Prism (version 9.2, Dotmatics).

## Results

### Participant characteristics

A total of 298 subjects aged ≥ 60 years were recruited for the study, comprising 44 subjects with mGFR < 30 ml/min/1.73 m^2^, 163 subjects with mGFR 30–59 ml/min/1.73 m^2^, and 91 subjects with mGFR ≥ 60 ml/min/1.73 m^2^. Of the participants, 43.96% were female. The age, Scr, and CysC were 71 (65, 77) years, 116.00 (91.53, 168.83) μmol/L, and 1.48 (1.14, 2.01) mg/L, respectively. The mGFR was 48.77 (36.50, 62.45) ml/min/1.73 m^2^. Detailed characteristics and eGFR values estimated by different equations are provided in Table 2.

**Table 2:** General characteristics of 298 Chinese subjects

Characteristic	Total	mGFR (ml/min/1.73 m^2^)			*P*
		< 30	30–59	≥ 60	
Number (*n*)	298	44	163	91	
Female (*n* [%])	131 (43.96%)	24 (54.55%)	68 (41.72%)	39 (42.86%)	0.312**
Age (years)	71 (65, 77)	72.20 ± 7.50	73 (67, 78)	68 (64, 73)	< 0.001*
Scr (μmol /L)	116.00 (91.53, 168.83)	317.5 (197, 464.5)	122.8 (101.6, 158.8)	87.3 (65.3, 102.2)	< 0.001*
Cysc (mg/L)	1.48 (1.14, 2.01)	3.07 (2.36, 3.6)	1.54 (1.31, 1.98)	1.02 (0.88, 1.32)	< 0.001*
mGFR (ml/min/1.73 m^2^)	48.77 (36.50, 62.45)	22.76 (18.02, 27.30)	45.00 (39.50, 51.30)	68.70 (63.40, 79.70)	< 0.001*
eGFR (ml/min/1.73 m^2^)					
CKD-EPI-Scr	50.38 (31.78, 66.97)	14.55 (9.34, 26.11)	43.98 (32.28, 56.59)	73.72 ± 18.89	< 0.001*
CKD-EPI-Cysc	49.08 (34.55, 68.60)	16.21 (12.45, 23.03)	40.20 (29.44, 51.92)	69.08 ± 21.05	< 0.001*
CKD-EPI-Scr-Cysc	45.28 (29.39, 63.97)	15.46 (10.43, 19.07)	41.36 (30.21, 51.80)	70.04 (56.09, 85.14)	< 0.001*
MDRD	51.65 (34.21, 68.84)	15.77 (10.47, 28.68)	46.68 (35.07, 59.96)	76.54 ± 20.41	< 0.001*
MDRDc	54.00 (34.62, 73.79)	15.36 (9.96, 27.94)	47.22 (35.78, 62.32)	77.25 (66.13, 101.71)	< 0.001*
FAS	38.73 (26.59, 50.93)	14.01 (9.93, 21.39)	36.68 ± 12.27	52.66 (46.04, 71.76)	< 0.001*
BIS1	48.80 (33.97, 60.52)	19.95 (14.20, 28.35)	44.18 ± 13.27	66.49 ± 14.64	< 0.001*

All data were collected from each individual in this study. Normally distributed data are presented as mean ± standard deviation ($\overline{x }$± s), and non-normally distributed data are presented as median (interquartile range [IQR])

*Scr* Serum creatinine, *Cysc* Cystatin C, *mGFR* measured glomerular filtration rate, *MDRD* Modification of diet in renal disease, *MDRDc* MDRD-China, *CKD-EPI* Chronic kidney disease epidemiology collaboration, *BIS1* Berlin Initiative Study 1, *FAS* Full age spectrum. Reference range: Scr, 57–110 μmol/l in male and 53–97 μmol/l in female

*Non-parametric rank-sum test

**Chi-square test, comparing between subjects with different mGFR levels

### Comparisons of the applicability of the examined equations across all samples

The results of these analyses revealed that, except for the MDRD and MDRDc equations, the other five equations were more likely to underestimate mGFR (bias < 0) (Table 3). The bias of the MDRDc equation significantly differed from all other equations except the MDRD equation (*P* < 0.05), and the bias of the MDRD equation differed from the other four equations except the MDRD and CKD-EPI-Scr equations (*P* < 0.01). The bias of the FAS equation was apparently different from the other equations except the MDRD and MDRDc equations (*P* < 0.01). There was no significant difference in the bias of other equations (*P* > 0.05).

**Table 3:** Performance of the seven equations in the overall sample and in the subgroups

Items	Bias Median	Precision IQR	Accuracy P30 (30%)	RMSE
Overall samples				
CKD-EPI-Scr	– 0.29	18.21 (– 10.16,8.04)	62.4%	16.99
CKD-EPI-Cysc	– 3.85	14.58 (– 10.54,4.04)	67.1%	16.19
CKD-EPI-Scr-Cysc	– 3.26	18.44 (– 12.45,6.00)	66.4%	15.39
MDRD	2.23	19.29 (– 8.17,11.12)	64.1%	18.03
MDRDc	4.00	24.44 (– 7.79,16.65)	57.7%	21.33
FAS	– 9.46	16.04 (– 17.67,– .1.63)	59.7%	17.11
BIS1	– 2.02	14.61 (– 10.23,4.37)	72.1%	13.53
mGFR < 30 ml/min per 1.73 m^2^				
CKD-EPI-Scr	– 5.24	13.58 (– 12.10,1.48)	38.6%	12.51
CKD-EPI-Cysc	– 4.56	6.77 (– 8.82,– 2.05)	59.0%	8.26
CKD-EPI-Scr-Cysc	– 5.65	9.23 (– 10.82,– 1.59)	43.6%	9.91
MDRD	– 3.71	14.91 (– 10.63,4.28)	43.2%	12.25
MDRDc	– 4.73	15.29 (– 11.30,3.99)	40.9%	13.06
FAS	– 5.94	10.74 (– 11.11,– 0.37)	38.6%	10.44
BIS1	– 0.96	11.71 (– 7.43,4.28)	47.7%	10.63
mGFR 30–59 ml/min/1.73 m^2^				
CKD-EPI-Scr	0.30	17.87 (– 9.92,7.95)	63.2%	16.47
CKD-EPI-Cysc	– 4.73	16.73 (– 13.13,3.60)	69.4%	13.92
CKD-EPI-Scr-Cysc	– 2.50	15.08 (– 10.82,4.26)	68.8%	13.96
MDRD	2.61	18.02 (– 7.88,10.14)	63.8%	17.22
MDRDc	4.46	22.07 (– 7.49,14.58)	60.7%	19.45
FAS	– 9.32	14.38 (– 16.01,– 1.63)	63.8%	14.35
BIS1	– 0.43	12.85 (– 7.90,4.95)	74.8%	11.92
mGFR ≥ 60 ml/min/1.73 m^2^				
CKD-EPI-Scr	2.25	22.92 (– 10.07,12.86)	72.5%	19.60
CKD-EPI-Cysc	– 3.57	33.29 (– 20.17,13.12)	66.7%	21.62
CKD-EPI-Scr-Cysc	– 0.33	26.69 (– 15.83,10.86)	72.2%	19.27
MDRD	4.92	23.70 (– 7.34,16.37)	74.7%	21.48
MDRDc	8.49	30.70 (– 4.60,26.10)	60.4%	27.02
FAS	– 15.83	20.29 (– 24.81,– 4.52)	62.6%	23.19
BIS1	– 6.72	17.88 (– 15.89,1.99)	79.1%	17.04

Bias = eGFR-mGFR

*Scr* Serum creatinine, *Cysc* Cystatin C, *MDRD* Modification of diet in renal disease, *MDRDc* MDRD-China, *CKD-EPI* Chronic kidney disease epidemiology collaboration, *BIS1* Berlin Initiative Study 1, *FAS* Full age spectrum, *IQR* Interquartile range, *P30* Percentage of estimates within 30% of the measured value, *RMSE* Root-mean-square error

Regarding IQR results, the MDRDc equation had the highest IQR value, followed by the MDRD equation. The IQR values of the remaining equations were arranged in descending order as the CKD-EPI-Scr-Cysc, CKD-EPI-Scr, FAS, BIS1, and CKD-EPI-Cysc equations. The MDRDc and FAS equations showed accuracies (P30) lower than 60% (57.7% and 59.7%, respectively) and were significantly lower than other equations (*P* < 0.05). The accuracy (P30) of the BIS1 Eq. (72.1%) was significantly higher than all other tested equations (*P* < 0.05), while the accuracy (P30) of the remaining four equations (CKD-EPI-Scr 62.4%, CKD-EPI-Cysc 67.1%, CKD-EPI-Scr-Cysc 66.4%, MDRD 64.1%) did not differ significantly from each other (*P* > 0.05).

**Fig. 2:**
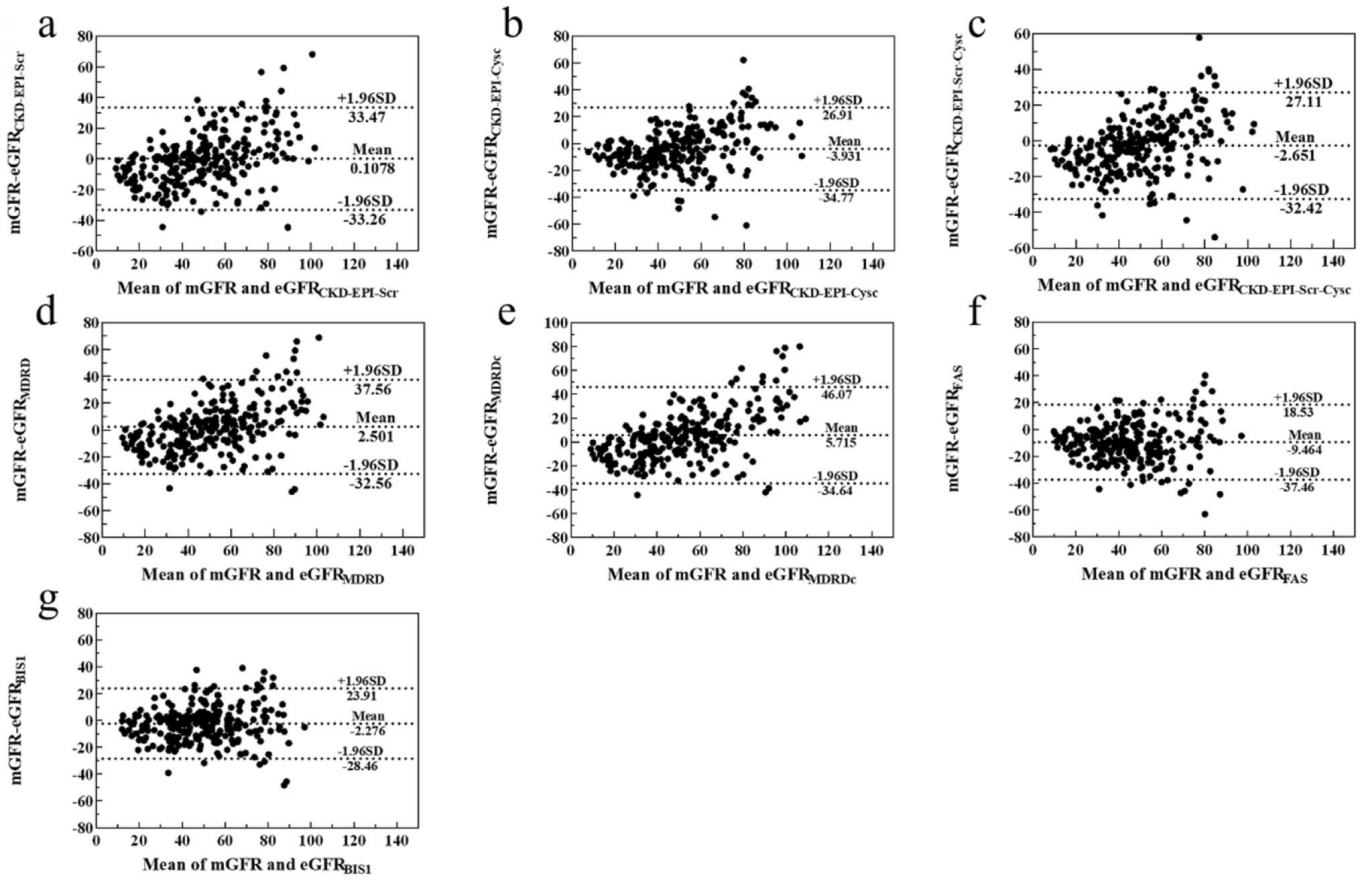
Comparisons of mGFR and eGFR. **a** CKD-EPI-Scr equation; **b** CKD-EPI-Cysc equation; **c** CKD-EPI-Scr-Cysc equation; **d** MDRD equation; **e** MDRDc equation; **f** FAS equation; **g** BIS1 equation. Solid and dashed lines in the Bland–Altman plot represent the mean and 95% limits of agreement (LoA) of difference, respectively. *CKD-EPI* chronic kidney disease epidemiology collaboration, *MDRD* modification of diet in renal disease, *MDRDc* MDRD-China, *FAS* full age spectrum, *BIS1* Berlin Initiative Study 1

The RMSE of the BIS1 equation was the smallest, while the RMSE of the MDRD equation was the largest, with the RMSE of the other equations fluctuating around 15–18. The Bland–Altman plots analysis demonstrated that the BIS1 equation exhibited the highest accuracy, with 95% confidence interval (CI) widths of 52.37. Meanwhile, FAS, CKD-EPI-Scr-Cysc, CKD-EPI-Cysc, and CKD-EPI-Scr equations exhibited slightly lower accuracies with 95% CI widths of 55.99, 59.53, 61.68, and 66.73, respectively. The MDRD and MDRDc equations exhibited the lowest accuracy with 95% CI widths of 70.12 and 80.71 (Fig. 2, Table 4).

**Table 4:** Diagnostic accuracy comparison based on Bland–Altman plots analysis

Items	Mean difference	Acceptable limits (95% CI)
CKD-EPI-Scr	0.1078	66.73 (– 33.26,33.47)
CKD-EPI-Cysc	– 3.931	61.68 (– 34.77,26.91)
CKD-EPI-Scr-Cysc	– 2.651	59.53 (– 32.42,27.11)
MDRD	2.501	70.12 (– 32.56,37.56)
MDRDc	5.715	80.71 (– 34.64,46.07)
FAS	– 9.464	55.99 (– 37.46,18.53)
BIS1	– 2.276	52.37 (– 28.46,23.91)

*CI* confidence interval, *Scr* Serum creatinine, *Cysc* Cystatin C, *MDRD* Modification of diet in renal disease, *MDRDc* MDRD-China, *CKD-EPI* Chronic kidney disease epidemiology collaboration, *BIS1* Berlin Initiative Study 1, *FAS* Full age spectrum

### Comparisons of the applicability of the examined equations for different mGFR level groups

All subjects were divided into three groups based on their mGFR levels: the mGFR < 30 ml/min/1.73 m^2^ group, the mGFR 30 ~ 59 ml/min/1.73 m^2^ group, and the mGFR ≥ 60 ml/min/1.73 m^2^ group.

For the mGFR < 30 ml/min/1.73 m^2^ group, there was no significant difference in bias among the seven equations (*P* > 0.05). IQR results revealed that the CKD-EPI-Cysc equation had the lowest IQR value (6.77), while the MDRDc equation had the highest IQR value (15.29). The accuracy (P30) of the CKD-EPI-Cysc and BIS1 equations was the highest (59.0% and 47.7%, respectively) but did not significantly differ from the rest of the equations (CKD-EPI-Scr 38.6%, CKD-EPI-Scr-Cysc 43.6%, MDRD 43.2%, MDRDc 40.9%, FAS 38.6%, *P* > 0.05). In this group, the accuracy of all equations was below 60%. The RMSE results showed that the CKD-EPI-Cysc and CKD-EPI-Scr-Cysc equations had the lowest values, followed by the FAS and BIS1 equations, while the remaining three equations had values greater than 12 (Table 3).

In the mGFR 30 ~ 59 ml/min/1.73 m^2^ group, the bias of the FAS equation was significantly lower than the bias of the other tested equations (*P* < 0.05), except for the CKD-EPI-Cysc equation (*P* > 0.05). The bias of the MDRD and MDRDc equations was positive and significantly different from the CKD-EPI-Cysc and CKD-EPI-Scr-Cysc equations (*P* < 0.05). The bias of the other equations did not significantly differ (*P* > 0.05). IQR results indicated that the BIS1 and FAS equations had the lowest values (12.85 and 14.38, respectively), while the MDRDc and MDRD equations had the highest values (22.07 and 18.02, respectively). The MDRDc equation also showed the lowest accuracy (P30 60.7%), while the BIS1 equation showed the highest accuracy (P30 74.8%). However, the accuracy of the equations did not significantly differ (P30 CKD-EPI-Scr 63.2%, CKD-EPI-Cysc 69.4%, CKD-EPI-Scr- Cysc 68.8%, MDRD 63.8%, FAS 63.8%, *P* > 0.05). Similar results were obtained for RMSE, with the BIS1 equation exhibiting the lowest RMSE and the MDRDc equation having the highest RMSE (Table 2).

In the mGFR ≥ 60 ml/min/1.73 m^2^ group, the bias of the FAS equation was significantly smaller than the other examined equations (*P* < 0.05), except for the BIS1 equation. The bias of the MDRD and MDRDc equations was the highest; the bias of the MDRD equation was significantly higher than the BIS1 equation (*P* < 0.05), and the bias of the MDRDc equation was notably higher than the CKD-EPI-Cysc, CKD-EPI-Scr-Cysc, and BIS1 equations (*P* < 0.05). The bias of the remaining equations did not differ significantly (*P* > 0.05). The BIS1 equation had the lowest IQR value, followed by the FAS, CKD-EPI-Scr, MDRD, CKD-EPI-Scr-Cysc, and MDRDc equations, while the CKD-EPI-Cysc equation had the highest IQR value. Among the tested equations, the accuracy (P30) of the BIS1 equation was as high as 79. 1%, the accuracy of the MDRD, CKD-EPI-Scr, and CKD-EPI-Scr-Cysc equations was also above 70% (MDRD 74.7%, CKD-EPI-Scr 72.5%, CKD-EPI-Scr-Cysc 72.2%), and the accuracy of the remaining three equations fluctuated between 60 and 70% (CKD-EPI-Cysc 66.7%, MDRDc 60.4%, FAS 62.6%). However, the differences in accuracy between these equations were not significant (*P* > 0.05). The RMSE results revealed that BIS1, CKD-EPI-Scr-Cysc, and CKD-EPI-Scr equations had RMSE values lower than 20, the CKD-EPI-Cysc and MDRD equations had slightly increased RMSE values, and the FAS and MDRDc equations had the highest RMSE values.

## Discussion

GFR is of paramount importance in drug dosing, CKD diagnosis, treatment, and prognosis evaluation. Direct measurement of GFR using ^99m^ Tc-DTPA clearance has been shown to correlate well with inulin clearance [18]. In our research, we also used ^99m^ Tc-DTPA renal dynamic imaging as a reference. However, ^99m^ Tc-DTPA renal dynamic imaging is not widely available in many grassroots medical institutions in China, and the cost of this examination remains a significant economic burden for numerous patients. Consequently, developing an accurate and reliable method for estimating GFR is essential. In this study, we compared the effectiveness of seven commonly used eGFR equations in terms of bias, accuracy, and precision.

Currently, the CKD-EPI and MDRD equations are the two most frequently recommended equations for adults [21, 22]. The FAS equation, developed based on 6870 patients from Europe and the United States (including 1764 elderly individuals over 70 years old), is applicable to all age groups [15]. These equations are based on Scr levels or Cysc levels and are not specifically designed for the elderly population. However, muscle mass, protein intake, and the prevalence of chronic diseases vary among different age and ethnic groups, affecting creatinine levels [23]. Cysc is less influenced by these factors, but the elderly are underrepresented in the development and validation of Cysc-based equations [13, 24]. The BIS1 equation is tailored for individuals aged 70 and above; however, the samples included in the equation's development were all residents of Berlin [14]. Thus, the applicability of these equations to the elderly in China remains uncertain. Our research aims to shed light on the accuracy and precision of these seven equations in the Chinese population aged 60 and above, presenting different glomerular filtration rates.

In our study, the BIS1 equation demonstrated the highest accuracy and the smallest RMSE among overall subjects and in mGFR ≥ 30 ml/min/1.73 m^2^ groups. This finding aligns with the BIS1 developer's assertion that the BIS1 equation is more accurate in elderly patients with normal renal function or only moderate decline [14]. According to the K/DOQI guideline, the acceptable level of P30 for GFR prediction equations should exceed 70% [19]; the BIS1 equation was the only one of the seven equations that met this standard in overall subjects. The Bland–Altman diagram also indicated that the BIS1 equation had the narrowest acceptable limits.

When mGFR falls below 30 ml/min/1.73 m^2^, the applicability of all equations was unsatisfactory, as the highest accuracy was only 59%, and all equations underestimated the true GFR. Consequently, for the elderly population, when eGFR drops below 30 ml/min/1.73 m^2^, it is advisable to measure GFR or evaluate the patient's condition in conjunction with their clinical symptoms. Otherwise, there is a risk of misjudging the severity of the patient's condition, exacerbating their anxiety, and even prompting premature renal replacement therapy. When mGFR is between 30 and 59 ml/min/1.73 m^2^, only the BIS1 equation achieves 70% accuracy, and its precision is superior to the other six equations. When mGFR ≥ 60 ml/min/1.73 m^2^, the accuracy of the CKD-EPI-Scr, CKD-EPI-Scr-Cysc, MDRD, and BIS1 equations all met the standard, but the BIS1 equation's performance remained the best, boasting the lowest IQR and RMSE and the highest accuracy. Therefore, we can conclude that the BIS1 equation is the most promising for use in the Chinese elderly population with mGFR ≥ 30 ml/min/1.73 m^2^.

In a cohort of older adults from Iceland, Li Fan evaluated various equations for estimating the prevalence of GFR reduction (mGFR < 60 ml/min/1.73 m^2^) and their performance in estimating mGFR. They discovered that the CKD-EPI equations had similar or better performance than the BIS1 equation within the measurement range of the test [25]. Other studies comparing the CKD-EPI and BIS equations in the elderly have reported generally comparable performance, although some differences exist among studies [26–28]. However, since Asians constitute a relatively small proportion of the CKD-EPI equations' development population [12], the performance of the CKD-EPI equations in Chinese older individuals is not as robust as in the aforementioned studies.

### Limitations of the study

In this study, there are several limitations that should be acknowledged. First, our research was conducted in a single-center setting, which may not fully represent the diverse elderly population in China. Second, it is important to consider the significant impact of diabetes and hypertension on CKD patients when evaluating the applicability of each equation. A more detailed analysis could be performed by stratifying patients according to their primary diseases. Lastly, serum values in this study were measured only once for each subject, which could potentially affect the accuracy of the results.

## Conclusion

In this study, we aimed to assess the applicability of various GFR estimation equations, including the CKD-EPI series, MDRD, MDRDc, FAS, and BIS1, in the context of the elderly Chinese population. Our findings revealed that when mGFR was less than 30 ml/min/1.73 m^2^, all of the evaluated equations demonstrated suboptimal performance, indicating a need for further refinement or alternative approaches in this specific range of renal function.

On the other hand, for subjects with mGFR values greater than or equal to 30 ml/min/1.73 m^2^, our analysis showed that the BIS1 equation exhibited the most superior performance among the tested equations. This finding underscores the potential value of the BIS1 equation in estimating GFR for elderly Chinese individuals with mGFR levels over 30 ml/min/1.73 m^2^. As a result, healthcare professionals and researchers could consider the BIS1 equation as a more suitable and reliable method for GFR estimation in this population, which may ultimately contribute to improved diagnosis, treatment, and management of renal diseases among elderly Chinese patients.

## Data Availability

The dataset supporting the conclusions of this article is included within the article. The datasets generated and/or analyzed during the current study are available from the corresponding author upon reasonable request.

## Abbreviations

GFR: Glomerular filtration rate
eGFR: Estimated GFR
Scr: Serum creatinine
Cysc: Serum cystatin C
mGFR: Measured GFR
IQR: Interquartile range
P30: Percentage of estimates within 30% of the measured value
RMSE: Root-mean-square error
CI: Confidence interval
CKD: Chronic kidney disease
MDRD: Modification of diet in renal disease
MDRDc: MDRD-China
KDIGO: Kidney Disease/Improving Global Outcomes
CKD-EPI: Chronic Kidney Disease Epidemiology Collaboration
BIS: Berlin Initiative Study
FAS: Full age spectrum
^99^^m^ Tc-DTPA: 99M-Technetium-diethylenetriamine-pentaacetic acid

## Declarations

### Data transparency

All authors agreed with the content and all gave explicit consent to submit and that they obtained consent from the responsible authorities at the institute/organization where the work has been carried out, before the work is submitted.

### Research resource identifiers (RRID)

Software: IBM SPSS Version 26.0 RRID: SCR_016479. GraphPad Prism version 9.2 RRID: SCR_002798.
